# Characterization of Bacteriophages Infecting Clinical Isolates of *Clostridium difficile*

**DOI:** 10.3389/fmicb.2018.01701

**Published:** 2018-07-31

**Authors:** Wichuda Phothichaisri, Puey Ounjai, Tanaporn Phetruen, Tavan Janvilisri, Pongsak Khunrae, Sombat Singhakaew, Piyada Wangroongsarb, Surang Chankhamhaengdecha

**Affiliations:** ^1^Department of Biochemistry, Faculty of Science, Mahidol University, Bangkok, Thailand; ^2^Department of Biology, Faculty of Science, Mahidol University, Bangkok, Thailand; ^3^Department of Microbiology, Faculty of Science, King Mongkut's University of Technology Thonburi, Bangkok, Thailand; ^4^Department of Medical Sciences, National Institute of Health, Ministry of Public Health, Nonthaburi, Thailand

**Keywords:** *Clostridium difficile*, temperate phage, phage induction, host range, *Myoviridae*

## Abstract

*Clostridium difficile* is recognized as a problematic pathogen, causing severe enteric diseases including antibiotic-associated diarrhea and pseudomembranous colitis. The emergence of antibiotic resistant *C. difficile* has driven a search for alternative anti-infection modalities. A promising strategy for controlling bacterial infection includes the use of bacteriophages and their gene products. Currently, knowledge of phages active against *C. difficile* is still relatively limited by the fact that the isolation of phages for this organism is a technically demanding method since bacterial host themselves are difficult to culture. To isolate and characterize phages specific to *C. difficile*, a genotoxic agent, mitomycin C, was used to induce temperate phages from 12 clinical isolates of *C. difficile*. Five temperate phages consisting of ΦHR24, ΦHN10, ΦHN16-1, ΦHN16-2, and ΦHN50 were successfully induced and isolated. Spotting assays were performed against a panel of 92 *C. difficile* isolates to screen for susceptible bacterial hosts. The results revealed that all the *C. difficile* phages obtained in this work displayed a relatively narrow host range of 0–6.5% of the tested isolates. Electron microscopic characterization revealed that all isolated phages contained an icosahedral head connected to a long contractile tail, suggesting that they belonged to the *Myoviridae* family. Restriction enzyme analysis indicated that these phages possess unique double-stranded DNA genome. Further electron microscopic characterization revealed that the ΦHN10 absorbed to the bacterial surface via attachment to cell wall, potentially interacting with S-layer protein. Bacteriophages isolated from this study could lead to development of novel therapeutic agents and detection strategies for *C. difficile*.

## Introduction

Antimicrobial resistance has become one of the most serious global healthcare problems. The world mortality rate from drug resistant bacteria is estimated to be 700,000 per year and if these trends continue, by 2050, 10 million deaths a year will be reached (O'Neill and The Review on Antimicrobial Resistance, 2014). A Gram-positive, spore-forming bacterium, *C. difficile* is considered one of the most important drug resistant pathogens (Harnvoravongchai et al., 2017) and has been listed by the Centers for Disease Control and Prevention as an urgent threat. This anaerobic, toxin-producing bacterium is the leading cause of antibiotic-associated diarrhea in nosocomial setting (Rineh et al., 2014). *C. difficile* infection (CDI) can result in a wide range of clinical symptoms from asymptomatic carrier, mild diarrhea with low fever, colitis, pseudomembranous colitis to life-threatening fulminant colitis (Libby and Bearman, 2009; Buffie et al., 2012). Current treatment options for CDI largely depend on specific antibiotic treatment combined with supportive care, which address the high rate of recurrence due to resistant trait development (Hedge et al., 2008). The most recent option for recurrent CDI treatment is fecal microbiota transplantation (FMT), by using stool collected from healthy donors to treat CDI patients. This treatment succeeds to treat dysbiosis patients with remarkable high efficacy rate up to 81% (Drekonja et al., 2015). Not only bacteria but also bacteriophages and other active components are transferred in FMT that can affect the treatment outcome (Zuo et al., 2017). Therefore, the screening and validation of stool FMT donors are challenging for this approach. However, the incidence, severity and recurrence rates of CDI have markedly risen in both hospital and community settings over the past two decades (Vindigni and Surawicz, 2015). Declining of CDI was reported in last few years, which is considered due to good control, management, and following to CDI prevention guideline (Evans et al., 2017). The change in *C. difficile* epidemiology may be caused by inappropriate antibiotic usage, emergence of hypervirulent strains and outbreaks of CDI in hospitals (Aslam et al., 2005). Therefore, to mitigate the severity of CDI outbreaks and reduce the disease recurrence, alternative approaches for effective control of CDI are urgently needed (Joerger, 2003; Rea et al., 2013).

Bacteriophages pose one of the promising alternatives to be used in a control of resistant bacteria (Lin et al., 2017). Phages can be highly specific to their bacterial hosts. Many of the phages exhibit a very narrow host range. They can target, degrade and penetrate into bacterial biofilm and form plaques (Parasion et al., 2014; Nale et al., 2016). These characteristics highlight their potentials to be utilized as novel therapeutics for antibiotic resistant bacterial infections (Viertel et al., 2014). The possibility of using phages for curing bacterial infections was first described by Felix d' Herelle since the early 1900's (Wilkinson, 2001). However, after the advent of antibiotics in the 1940s, less attention was paid to research in phage therapy (Abedon et al., 2011; Viertel et al., 2014; Lin et al., 2017). The interest in utilizing phages as antibacterial agents has recently re-emerged due to rapid increase in antibiotic resistance and decline in novel antibiotic discovery (Ventola, 2015; Lin et al., 2017). Recent advances in molecular and sequencing techniques opens a tremendous opportunity to engineer phages and phage-derived proteins for therapeutic applications.

Phages may adopt two alternative life cycles, which are lytic or lysogenic. The lytic phages kill the host bacteria by causing cell lysis, while the temperate phages are able to insert their genome into the host in their lysogenic life cycle, forming prophages and replicate with the host cell. However, temperate phages often switch back to lytic life cycle and break out from the host under stress condition (Lamont et al., 1993; Orlova, 2012). Previous reports have shown phages specific to *C. difficile* from environmental and clinical samples and demonstrated that all of the isolated phages did not follow a strictly lytic lifestyle, despite their lytic activity, as their genomes encode integrases (Goh et al., 2005; Govind et al., 2006; Meessen-Pinard et al., 2012; Sekulovic et al., 2014).

The interaction of bacteriophage and host is always initiated through the docking of phage particle to the corresponding receptors on host surface. Many components found on the bacterial surface were proposed to be phage receptors, including cell wall teichoic acid, cell surface glycoprotein, S-layer proteins, pili, and lipopolysaccharides (Larson et al., 1978; Rakhuba et al., 2010). Due to limited resources on *C. difficile* phages, the receptor of *C. difficile* phages has not yet been identified.

In this work, we isolated and established a library of phages from clinical *C. difficile* isolates. The morphology, host range analysis and pattern of their genetic materials of the *C. difficile* phages were characterized. Further investigations on the interaction between *C. difficile* and ΦHN10 phage suggested that S-layer protein could serve as a potential receptor of this phage in this bacterium. A library of phages established in this work could potentially provide fruitful information that could lead to further development of alternative treatment and detection for CDI.

## Materials and methods

### Bacterial cell culture and growth condition

Seventy-three clinical *C. difficile* isolates were used for prophage carriage determination and phage isolation. Twenty-six of them were previously isolated from *C. difficile* toxin-positive fecal samples of diarrheal patients admitted to Ramathibodi Hospital, Thailand, during 2010–2011 (Chankhamhaengdecha et al., 2013), and additional 47 strains were obtained from the National Institute of Health (NIH), Thailand. The isolates have previously been identified into 16 PCR ribotypes using agarose gel-based PCR ribotyping (Table 1). Nineteen *C. difficile* reference ribotypes used in this study were kindly provided by Prof. Nigel Minton, University of Nottingham. All isolates were routinely grown in pre-reduced brain heart infusion broth (BHI) supplemented with 1% sodium taurocholate. Cultures were incubated at 37°C in an anaerobic chamber (10% H_2_, 10% CO_2_, and 80% N_2_) (Don Whitley Scientific, West Yorkshire, UK).

**Table 1:** *C. difficile* strains used in this work and prophage content.

**Strain^a^**	**PCR ribotype^b^**	**PCR amplification of phage genes in group:**		**Induction with mitomycin C**		**Origin**	**Strain^a^**	**PCR ribotype^b^**	**PCR amplification of phage genes in group:**		**Induction with mitomycin C**		**Origin**
		**Myo**	**Sipho**	**Myo**	**Sipho**				**Myo**	**Sipho**	**Myo**	**Sipho**	
Std001	001	+	−	**ND**		Reference Strains	HN1	NT-15	−	−	−	−	Clinically relevant strains, NIH
Std017	017	+	−				HN2	017	+	−	−	−	
Std020	020	+	−				HN3	017	+	−	−	−	
Std023	023	+	−				HN4	017	+	−	−	−	
Std027	027	−	−				HN5	017	+	−	−	−	
Std029	029	+	−				HN6	017	+	−	−	−	
Std046	046	+	−				HN7	017	+	−	−	−	
Std053	053	+	−				HN8	NT-16	+	+	−	+	
Std056	056	+	+				HN9	017	+	−	−	−	
Std070	070	−	+				HN10	017	+	+	−	+	
Std075	075	+	−				HN11	017	+	−	−	−	
Std077	077	+	−				HN13	NT-16	+	+	−	+	
Std081	081	+	−				HN16	017	+	+	+	+	
Std095	095	+	+				HN17	NT-16	+	+	−	−	
Std117	117	+	−				HN18	NT-16	+	+	−	−	
Std126	126	−	−				HN19	017	+	−	−	−	
Std131	131	+	+				HN20	017	+	−	−	−	
R20291	027	+	−				HN21	017	+	+	-	+	
630	012	+	−				HN22	NT-15	+	−	−	−	
HR1	NT-1	−	−	−	−	Faecal samples of diarrheal patients, Ramathibodi Hospital	HN23	NT-15	+	−	−	−	
HR2	NT-2	+	+	+	+		HN24	NT-15	−	−	−	−	
HR13	NT-11	+	−	+	−		HN25	NT-15	+	−	−	−	
HR24	NT-7	+	−	+	−		HN26	NT-15	+	−	−	−	
HR27	NT-7	+	+	−	−		HN27	NT-15	+	−	−	−	
HR29	NT-11	−	+	−	+		HN28	NT-15	+	+	−	−	
HR31	NT-6	+	−	+	−		HN29	NT-15	+	+	−	−	
HR37	NT-12	+	+	+	−		HN30	NT-15	+	+	+	−	
HR38	NT-3	+	−	−	−		HN31	NT-15	+	−	+	−	
HR39	020	+	−	−	−		HN32	NT-15	+	−	−	−	
HR44	017	+	+	+	−		HN33	NT-15	+	−	+	−	
HR49	017	+	+	−	−		HN34	NT-17	+	−	−	−	
HR58	NT-8	−	−	−	−		HN35	NT-17	+	−	−	−	
HR59	NT-13	+	−	−	−		HN36	NT-15	+	−	+	−	
HR65	NT-9	−	−	−	−		HN37	NT-17	+	−	-	−	
HR67	NT-10	+	−	−	−		HN38	NT-17	+	−	+	−	
HR74	NT-4	+	+	−	−		HN39	NT-17	+	−	+	−	
HR91	NT-14	+	−	−	−		HN40	NT-15	+	−	−	−	
HR98	017	+	−	−	−		HN41	NT-17	+	−	−	−	
HR118	017	+	−	−	−		HN42	NT-17	+	−	−	−	
HR136	017	+	−	−	−		HN43	NT-17	+	−	−	−	
HR156	NT-5	-	−	−	−		HN44	NT-17	+	−	−	−	
HR163	017	+	−	−	−		HN45	NT-17	+	−	−	−	
HR166	NT-11	−	+	−	−		HN46	NT-17	+	−	−	−	
HR184	017	+	+	+	−		HN47	NT-17	+	−	−	−	
HR376	NT-3	−	−	−	−		HN48	NT-17	+	−	−	−	
							HN49	NT-17	+	−	−	−	
							HN50	017	+	+	+	+	

a,b *Data of strain isolation and PCR ribotyping are included in the accompanying paper (S. Wongkuna, N. Malaisree, A. Aroonnual, T. Janvilisri, D. Chotiprasitsakul, P. Chongtrakool, P. Wangroongsarb, S. Chankhamhaengdecha, submitted for publication). ND, not determined; NT, ribotype patterns not related to the standard ribotypes*.

### Identification of lysogenic strain from clinical *C. difficile* isolates

The presence of lysogenic strains in the clinical *C. difficile* strain collection was determined by PCR analysis. Two sets of degenerate primers targeting the *C. difficile* phage myovirus and siphovirus *holins* and the PCR amplification program were performed as described previously (Shan et al., 2012). Genomic DNA was extracted and purified from 73 clinical isolates of *C. difficile* using the E.Z.N.A. Bacterial DNA kit (Omega Bio-tek, GA, USA). The PCR products were analyzed through electrophoresis in 1.5% (w/v) agarose gels, stained with SYBR safe (Invitrogen), and visualized under UV radiation. The PCR products from 5 distinct *C. difficile* strains that were positive according to the amplification with these two sets of degenerate primers were sequenced to confirm that the amplified sequences were encoded by *C. difficile* phage *holins*.

### High-throughput induction and screening of prophages from lysogens

All lysogenic *C. difficile* strains were induced with mitomycin C using a high-throughput induction method. The method was modified from a previous protocol for rapid prophage induction from *Escherichia coli* (McDonald et al., 2010). Briefly, a 96-well plate containing BHI medium was inoculated with a single colony of lysogen and incubated at 37°C overnight under anaerobic condition. For phage induction, 10-fold dilution of overnight culture of each isolate in BHI medium was made into a fresh 96-well plate and incubated at 37°C for 8 h. Induction of phage was performed by adding 3 μg/ml mitomycin C to the culture with further incubation at 37°C for 12 h. Subsequently, the culture was diluted 10-fold. Phages were isolated using filtration of lysate through 0.22 μm filter (Merck Millipore, Germany). Isolated phages were then transferred into another 96-well plate. To get rid of host genomic DNA from the lysate, 5 μg/ml of DNaseI was then added to each well and incubated at 37°C for 1 h. The reaction was heat-inactivated at 99°C for 10 min. The completeness of digestion reaction was evaluated by PCR using bacterial *16s rRNA* gene specific primers, UFUL (5′-GCCTAACACATGCAAGTCGA-3′) and URUL (5′-CGTATTACCGCGGCTGCTG G-3′; (Khan et al., 2001)). The inducible temperate phage DNA in each heated lysates was confirmed by PCR using specific phage primers as described previously (Shan et al., 2012).

### Phage preparation

The strains of *C. difficile* containing inducible temperate phages, including HR24, HN10, HN16, and HN50, were chosen for large scale phage induction. Mitomycin C induction was performed on 500 ml of log phase *C. difficile* cultured in BHI broth supplemented with 10 mM MgCl_2_ and 10 mM CaCl_2_ under anaerobic condition. After 12 h induction, lysates were centrifuged at 12,000 × *g* for 30 min and filtered through 0.45 μm filter membrane (Merck Millipore). Phage lysates were concentrated using polyethylene glycol (PEG) precipitation as previously described (Sambrook and Russell, 2001). Briefly, the phage lysates were salted out in 1 M NaCl. After centrifugation at 12,000 × *g* for 10 min at 4°C, 10% PEG-8000/MgSO_4_ solution was added to supernatant fraction and incubated overnight. The samples were then pelleted down for 30 min at 12,000 × *g*. Pellets were dissolved in SM buffer (150 mM NaCl, Tris-HCl pH6.5, 10 mM MgCl_2_, 1 mM CaCl_2_). Equal volume of chloroform was then added to the phage solution and was centrifuged at 12,000 × *g* for 5 min to induce phase transition. The upper phase was collected. The concentrated phage was then screened for its sensitive host using spot test (Beck et al., 2009) on a panel of 92 different *C. difficile* isolates. The lawns of the indicator hosts were independently created by mixing *C. difficile* culture with 3 ml of 0.6% molten TYs agar (3% tryptose, 2% yeast extract) supplemented with 10 mM CaCl_2_ and 10 mM MgCl_2_. The mixture was immediately overlaid on 1.5% BHI agar. The overlay was allowed to set for 5 min. After that, 10 μl of concentrated phage was then spotted onto the overlay of different *C. difficile* isolates. Following the incubation at 37°C overnight, the sensitive host of each phage was determined based on the plaque formation.

To further isolate a single strain of phage, the double agar layer method was used (Gencay et al., 2017). Briefly, 1.5 ml of *C. difficile* sensitive host was mixed with 0.1 ml of serially diluted phage solution and incubated at 37°C for 10 min. The mixture was subsequently transferred to 3 ml of 0.6% molten TYs (top). After that, the mixture was immediately overlaid on the preformed 1.5% BHI agar (bottom). The plate was allowed to sit at room temperature to let the soft agar in the top layer solidify. The plate was then incubated at 37°C overnight under anaerobic condition and was monitored for plaque formation. Single plaque of different sizes was collected and resuspended in SM buffer. Double agar layer method was repeated at least three times using the collected plaque suspension from the previous round until a single plaque morphology was achieved. The purified phage from the final round was then propagated in its sensitive host using liquid culture. Briefly, the sensitive host of each phage was grown in 100 ml of TYs broth at 37°C for 8 h under anaerobic condition. Subsequently, 1 ml of isolated phage lysate was added to the culture and incubated for 10 min, following by the addition of 100 ml of fresh TYs broth to the mixture. Following the overnight incubation, phage lysate was collected, filtered, and concentrated using PEG precipitation as described above. The enriched phage fraction was then further purified using CsCl gradient as previously described (Sambrook and Russell, 2001). Solid CsCl was added to phage solution to a final concentration of 0.75 g/ml. The mixture was centrifuged at 150,000 × *g* for 24 h at 4°C. The white-gray band representing phage particles were collected and dialyzed three times against 1 L of SM buffer at 4°C using 50 kDa dialysis tube (Spectrumlab, USA). The purified phage suspension was kept at 4°C for further experiment. In case of long-term phage storage, purified phage suspension was mixed with 7% (v/v) dimethyl sulfoxide (DMSO) and stored at −80°C.

### Transmission electron microscopy (TEM) and image analysis

A droplet of selected *C. difficile* phages including ΦHN10, ΦHN16-1, ΦHN16-2, ΦHN50, and ΦHR24 were deposited on a continuous formvar-carbon or formvar-coated air glow discharged grids. The specimen was negatively stained using 2% uranyl acetate prior visualization under Tecnai T20 G^2^ electron microscope operating at the accelerating voltage of 120 keV. Images were captured using a CCD camera (Gatan). Negative stain EM of *C. difficile* cells were also performed using 2% uranyl acetate (Ackermann, 2009). The images were visualized in a Tecnai T20 G^2^ electron microscope at 1–1.5 μm defocus and recorded using Gatan CCD camera. Image analysis and Fourier calculation were conducted in Digital Micrograph software.

### Host range determination

The host range of phages, ΦHN10, ΦHN16-1, ΦHN16-2, ΦHN50, and ΦHR24, was preliminary determined by spot test (Beck et al., 2009) on 92 different *C. difficile* isolates as described above. Positive spot tests were further confirmed by modified double agar layer method (Kropinski et al., 2009). One point five ml of *C. difficile* culture were mixed with 0.1 ml of serially diluted purified phage and incubated at 37°C for 10 min. The mixture was subsequently transferred to 3 ml of 0.6% molten TYs and immediately overlaid on the preformed 1.5% BHI agar and allowed soft agar in the top layer to solidify. The plate was then incubated at 37°C overnight under anaerobic condition. The plate was observed and the size and appearance of the plaques were noted. Plaque size was measure using Vernier caliper. Phage titer was calculated from the number of plaque formation.

### Evaluation of phage stability

Stability tests were performed on 4 isolated phages (ΦHN10, ΦHN16-1, ΦHN16-2, and ΦHN50, excluding ΦHR24 due to lack of its sensitive host). To evaluate the ability of phages to tolerate different pH conditions, isolated phage samples were assayed in TYs broth prepared at different pH, ranging from pH 2 to pH 11. The samples were incubated at 37°C for 3 h (Capra et al., 2006). After that, phage titers were determined using spot-titer assays. Ten-fold dilutions of phage (10^5^-10^10^ PFU/ml) were spotted on lawn of bacterial host to determine phage existence. To examine thermal stability of isolated phages, samples of phages were incubated at various temperatures in a water bath (25, 37, 50, 60, and 70°C). The samples were collected at 5, 30, 60, 120, and 180 min (Capra et al., 2004). The titers of phages at various conditions were enumerated using spot-titer assays (Beck et al., 2009). The 10 μl volumes of several sample dilutions were spotted on a single plate, incubating, and observing for plaques.

### Nucleic acid extraction and restriction profile analysis

Nucleic acids of the 4 isolated phages including ΦHN10, ΦHN16-1, ΦHN16-2, and ΦHN50 were extracted using modified phenol/chloroform method as described elsewhere (Moineau et al., 1994). Briefly, 3 ml of concentrated phages were treated with 50 μg/ml DNaseI and 50 μg/ml RNaseA (Sigma-Aldrich, UK) for 1 h at 37°C. The reaction was terminated by adding 0.5 volume of an SDS mixture (0.5 M Tris-HCl pH 9.0, 0.25 M EDTA, 2.5% SDS) and incubating at 65°C for 15 min. An equal volume of 25:24:1 phenol-chloroform-isoamyl alcohol (Merck, UK) was then added into the solution. The mixture was then vigorously mixed and centrifuged at 12,000 × *g* for 15 min at room temperature. The aqueous phase was collected. This phenol-chloroform-isoamyl alcohol extraction step was repeated for two times. Nucleic acids were then precipitated in ice-cold 70% ethanol. The pellet was resuspended in 50 μl of TE buffer pH 8 and stored at −20°C until use. The profiling of phage DNA was performed by treating isolated phage DNA with 12 restriction endonucleases including *Alu*I, *Dpn*I, *Acc*I, *Bam*HI, *Cla*I, *Eco*RI, *Eco*RV, *Hin*cII, *Hin*dIII, *Nde*I, *Nco*I, and *Pst*I, according to manufacturer's instructions. Restriction pattern were visualized using 1% agarose gel electrophoresis at 90 V for 1 h.

### Phage attachment and phage receptor prediction

Cells in 5 ml of late log phase culture of *C. difficile* HN2, sensitive host of ΦHN10 phage, were collected by centrifugation at 3,000 × *g* for 5 min. Defective bacteria lacking of pili and flagella and other cellular appendages were prepared using mechanical agitation method (Novotny et al., 1969). The cells were resuspended in 200 μl phosphate buffer saline (PBS) supplemented with 10 mM CaCl_2_, 10 mM MgCl_2_, thioglycolic acid (TGA), and 5% sucrose. To disrupt cellular projection including pili and flagellum, bacterial cell suspensions were vigorously agitated in a vortex mixer and then subjected to incubation in a sonication bath at room temperature for 20 min. Defective bacterial cells lacking pili and other cellular appendages were then isolated by centrifugation at 5,000 × *g* for 2 min. Pellets of defective bacteria were resuspended in 200 μl fresh BHI broth. Susceptibility of intact and defective *C. difficile* cells to ΦHN10 was characterized by a spot-titer assay (Beck et al., 2009). To determine phage titer that is susceptible to each host cell types, 10-fold serial dilutions of ΦHN10 phage were prepared in culture media from 10^5^ to 10^10^ PFU/ml. Then, 10 μl of each dilution of the phage were spotted on the double agar layer. The lowest phage concentration was quantified on the basis of the numbers of plaques. The putative phage receptor on bacterial host cell for phage binding was investigated using transmission electron microscope. Fourier transforms of the *C. difficile* S-layers were calculated using Gatan Digital Micrograph Software.

### Extraction of S-layer proteins

S-layer proteins (SLPs) was extracted from *C. difficile* as previously described (Calabi et al., 2001). Overnight culture of *C. difficile* HN2 was transferred to 50 ml fresh BHI medium and cultured until OD_600_ reach 0.5. Cells were harvested by centrifugation at 3,000 × *g* for 20 min. Then cells were washed once with PBS buffer and resuspended in 200 μl of 200 mM glycine pH 2.2. Cells were incubated at room temperature for 30 min before removing intact and cell debris by centrifugation at 16,000 × *g* for 15 min at 4°C. S-layer protein-containing supernatant was collected, the pH of the solution was adjusted to 7 with 2 M Tris-HCl pH 9 and stored at 4°C.

### Gel shift assay of S-layer protein

To investigate the interaction between isolated phage, ΦHN10 and S-layer proteins, 1 mg/ml of S-layer protein was preincubated with 0.1 mg/ml of ΦHN10 in PBS buffer containing 150 mM NaCl for 6 h. The mixture was then subjected to native-PAGE analysis using the running buffer consisted of 40 mM Tris pH 10, 40 mM acetate and 1 mM EDTA. The gel was stained with Coomassie to observe the shift of the S-layer protein.

## Results

### Prophage induction and phage morphology

Lysogenic strains within clinical isolates of *C. difficile* were determined by PCR targeting-holin genes of myovirus and siphovirus (Supplementary Figure 1). The PCR products were confirmed by DNA sequencing, which were further blasted against nucleotide database in National Center for Biotechnology Information (NCBI). The results confirmed that the amplified PCR products were *holin* genes of myovirus or siphovirus. The results revealed that 66/73 clinical *C. difficile* isolates were lysogenics. The inducibility in all of the 66 lysogenic strains was then investigated by mitomycin C using high-throughput induction and screening method. Twenty-two (32.8%) of temperate phages were mitomycin C inducible. Myovirus *holin* genes were found in the phage preparation of 15 bacterial isolates including HR13, HR24, HR31, HR37, HR44, HR184, HN10, HN23, HN30, HN31, HN33, HN36, HN38, HN39, and HN47. While 4 isolates including HR29, HN8, HN13, and HN21 were siphovirus positive and 3 isolates, HR2, HN16, and HN50, appeared to harbor both myo- and siphovirus as summarized in Table 1.

In this study, phages from 4 *C. difficile* isolates HN10, HN16, HN50, and HR24 were selected for further analyses. The phages induced from these isolates were assigned according to the names of their respective hosts as ΦHN10, ΦHN16, ΦHN50, and ΦHR24. Interestingly, two prophages with different sizes and morphological features could be induced from the strain HN16. These two phages were isolated against their sensitive host using double agar layer method. The phages were designated as ΦHN16-1 and ΦHN16-2 as shown in Table 2. All inducible temperate phages possessed long, non-flexible, and contractile tail, indicating that they are members of *Myoviridae* family, in the order of *Caudovirales*. The phages contained icosahedral capsid head with the size ranging from 45 to 70 nm in diameter and the contractile tail with the length ranging from 125 to 250 nm. In addition, phage tail-like particle structures (PT-LPs) were also observed in the lysate of the isolates HR37 and HN47. The overall structure appeared similar to a regular phage tail of myovirus, but without icosahedral head. The size of particle was ~100 nm long, 20 nm wide with 40 nm horizontal cap at the one end (Table 2).

**Table 2:** Morphological characteristic and specific host of isolated phage in the family of Myoviridae.

**Phage**	**Specific host**	**Plaque appearance, diameter (mm)**	**Capsid (nm)**	**Tail length/tail width (nm)**	**Phage morphology**
ϕHR24	None	None	~70	~140/20	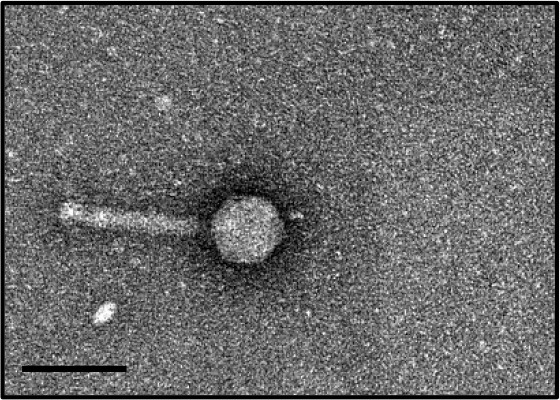
ϕHN10	630, HR118, HN2, HN6, HN9, HN21	Clear, 1.1^a^	~70	~250/23	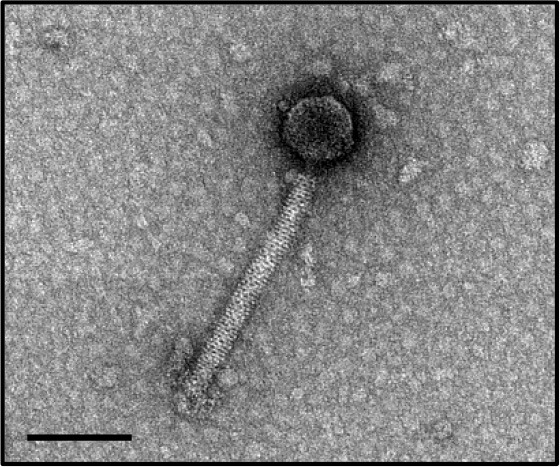
ϕHN16-1	HN21	Clear, 0.6	~45	~130/15	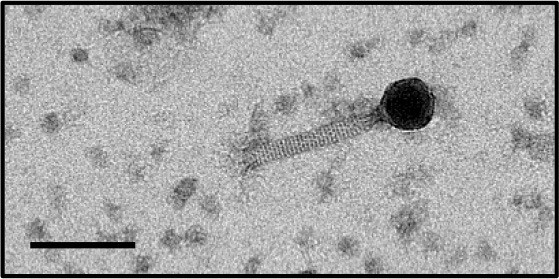
ϕHN16-2	HN21	Clear, 1.1	~60	~125/20	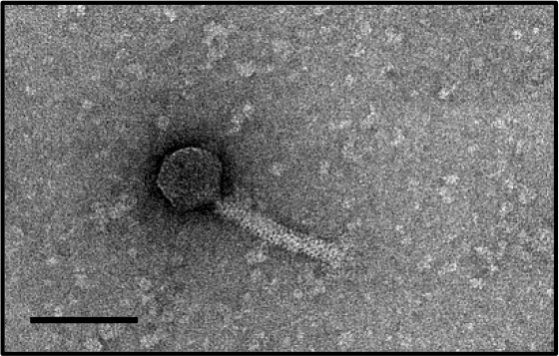
ϕHN50	HN21	Clear, 0.7	~60	~135/22	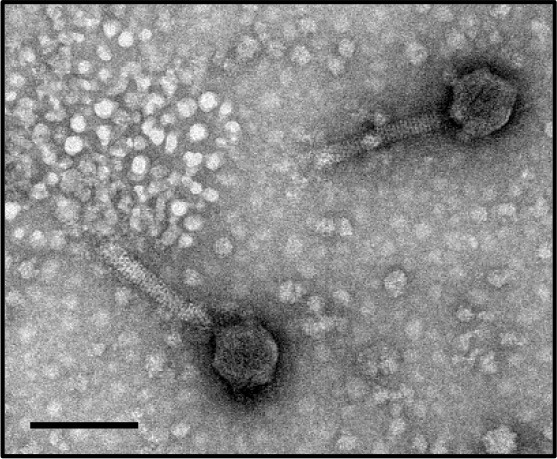
PT-LP	None	None	None	~100/20	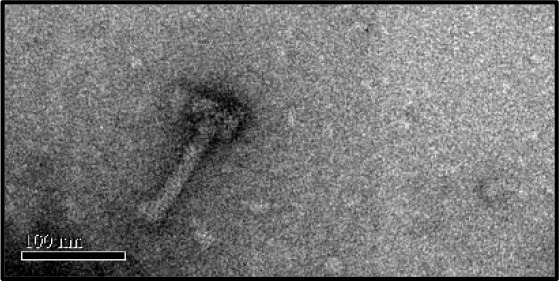

a *Plaque appearance and diameter were observed against all sensitive hosts and the same results were obtained*.

*PT-LP represented phage tail like particle. The phage images were taken with TEM at magnification of × 29,000. The black bar represented 100 nm*.

### Host range determination

In our study, the host ranges of the 5 *C. difficile* phages including ΦHN10, ΦHN16-1, ΦHN16-2, ΦHN50, and ΦHR24 were preliminary determined against a panel of 92 *C. difficile* strains containing standard ribotypes and clinical isolates by spot test. However, this test has been demonstrated to over-estimate the host range of a phage (Mirzaei and Nilsson, 2015). Therefore, sensitive hosts were confirmed using double agar layer method. Although no susceptible host was found for ΦHR24. The other 4 temperate *C. difficile* phages displayed a narrow host range against panel of indicator hosts tested. ΦHN10 produced clear plaques on 6 different isolates including 630, HR118, HN2, HN6, HN9, and HN21. The 630 strain is ribotype 012 whereas the remainder were ribotype 017. ΦHN16-1, ΦHN16-2, and ΦHN50, could only lyse HN21, which was ribotype 017 (Table 2).

### Stability of phages at different PH and temperature

**Figure 1:**
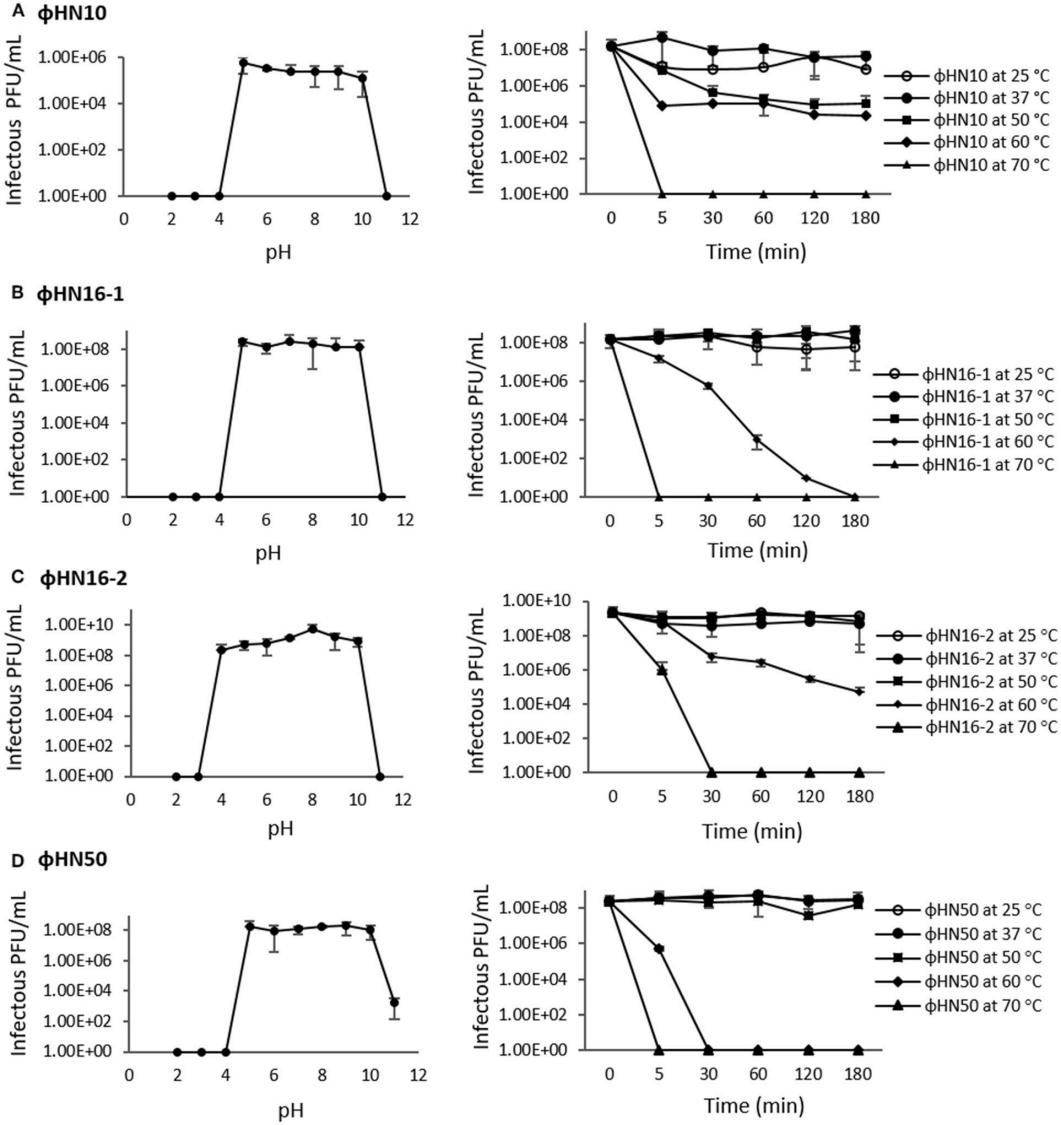
pH and thermal stability test of phages. The first panel represented phage infectivity after incubation at different pH ranging from pH 2 to pH 11 at 37°C for 180 min. The second panel represented phage infectivity after treated with different temperature as indicated time point. Phage titer were enumerated by spot-titer assay. **(A–D)** represent phages ΦHN10, ΦHN16-1, ΦHN16-2, and ΦHN50, respectively.

The pH stability of phage was carried out at different pH range from pH 2 to pH 11. After 180 min incubation, phages in this study were shown to have stable infectivity at wide ranges of pH from pH 5 to pH 10. While, the phages ΦHN10, ΦHN16-1, and ΦHN50 completely lost their infectivity at pH 2–4, ΦHN16-2 still maintained their activity at pH 4. Most of the 4 phages appeared to lose lytic activity at pH 11, except ΦHN50, which a significant decrease in activity was observed (Figure 1). The phage thermal tolerance was tested showing that ΦHN16-1, ΦHN16-2, and ΦHN50 had stable infection after incubation at 25, 37, and 50°C for 180 min and the phage infectivity was dramatically decreased at 60°C. More than 50% of ΦHN10 was still active after incubation at 50 and 60°C for 180 min. At 70°C, all phage particles lose their infectivity (Figure 1).

### Genomic restriction profile

**Figure 2:**
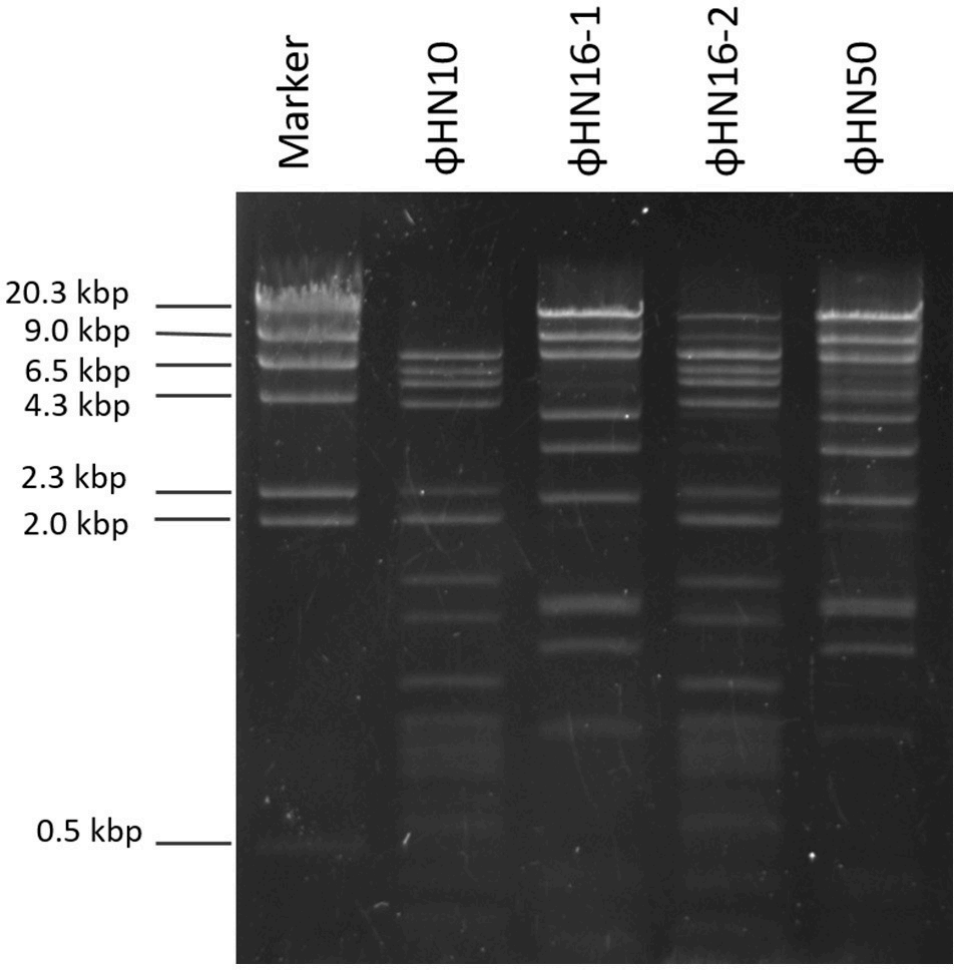
Genomic DNA from phages were digested with the *Hind*III enzyme and analyzed on 1% agarose gel. The stained gel showed separated fragment of ΦHN10, ΦHN16-1, ΦHN16-2, and ΦHN50. Lambda DNA digested with *Hind*III enzyme was included as a marker.

The DNA restriction profiles of 4 phages including ΦHN10, ΦHN16-1, ΦHN16-2, and ΦHN50, were examined. They exhibited distinct DNA restriction patterns following *Hin*dIII digestion, as shown in Figure 2. Thus, all the 4 isolated phages are considered different phages.

### Phage attachment on bacterial host

**Figure 3:**
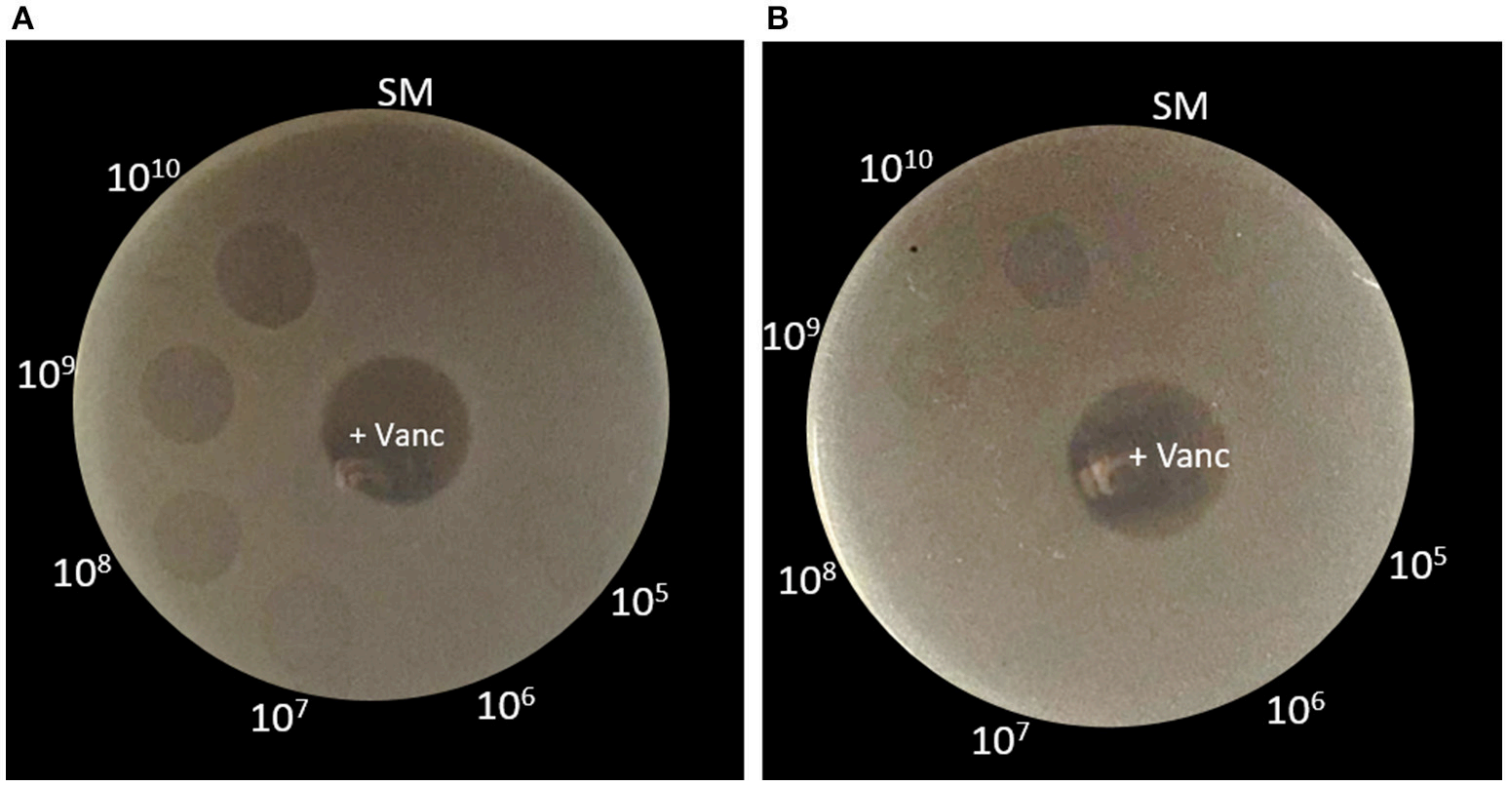
Spot-titer assay showed difference in phage susceptibility between normal and defective bacterial hosts. **(A)** For normal HN2 cells, they were sensitive to phage at 10^5^ PFU/ml. **(B)** Defective HN2 cells were sensitive to phage at 10^9^ PFU/ml. +Vanc, Vancomycin as positive control; SM, buffer as negative control.

**Figure 4:**
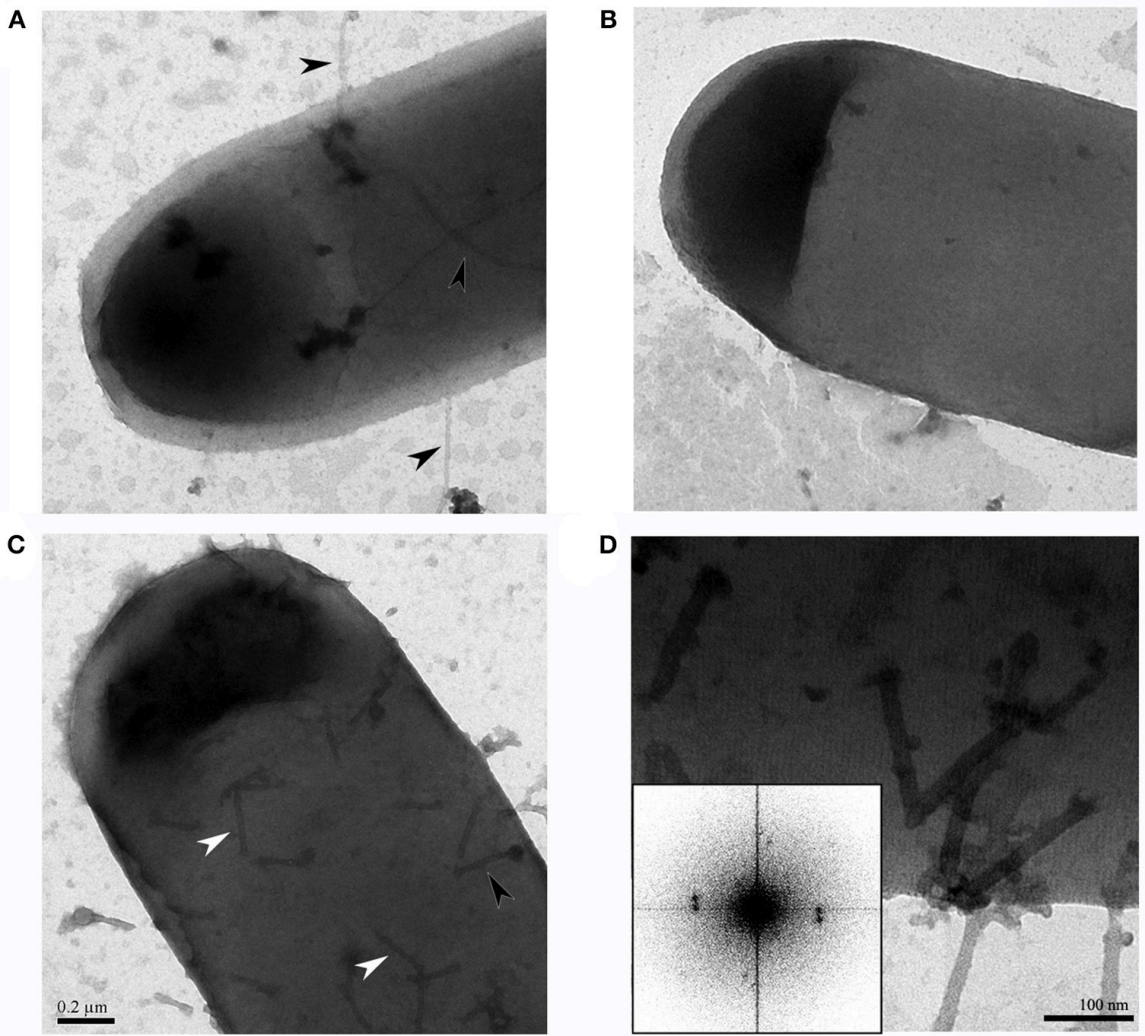
TEM images revealed the binding site of *C. difficile* phages on the cell, **(A)** Intact *C. difficile* HN2 isolate contained pili as indicated by black arrowheads, **(B)** After mechanical disruption, *C. difficile* HN2 isolate showed absence of pili, **(C)** Intact and ghost particles of ΦHN10 phages attach on mechanical disrupted pili-less *C. difficile* host as depicted by black and white arrowheads respectively, scale bar for **(A–C)** is 200 nm **(D)** Expanded view of phage attached pili-less *C. difficile* revealed that phages adhered the to a mesh-like structure on the cell surface. Inset: Fourier transform pattern of the phage attached pili-less cell confirmed the existence of well-ordered S-layer of *C. difficile* with tetragonal lattice (Cerquetti et al., 2000), scale bar in **(D)** represented 100 nm.

To investigate the potential phage receptor on bacterial cells, mechanical force was used to disrupt pili and part of bacterial cell surface from *C. difficile* HN2. It showed lower susceptibility to phage infection after mechanical treatment. In normal condition, HN2 host could be lysed by phage at 10^5^ PFU/ml, while defective HN2 host could be lysed by higher concentration of phage at 10^9^ PFU/ml (Figure 3). The results suggested that ΦHN10, as a representative *Myoviridae* phage, exhibited lower efficiency in infection to their defective host. Identification of putative phage receptor was performed using TEM analysis. Normal *C. difficile* HN2 cells possessed pili (Figure 4A), whereas the pili are absent in the mechanically treated cells (Figure 4B). Moreover, the treated cells appear to have thinner cell wall when compared with the untreated cells. Despite, having no pili, abundant binding of phages was observed on the cells of treated bacteria. The particle appeared to bind to a mesh-like structure on bacterial cell surface (Figure 4C). To determine the pattern of surface array, the phage binding areas in the images were subjected to Fourier analysis. The Fourier image revealed tetrahedral arrangement of the subunits (Figure 4D), suggesting that the mesh-like structure on *C. difficile* that binds to the phages is likely to be an S-layer.

### Identification of phage-receptor

**Figure 5:**
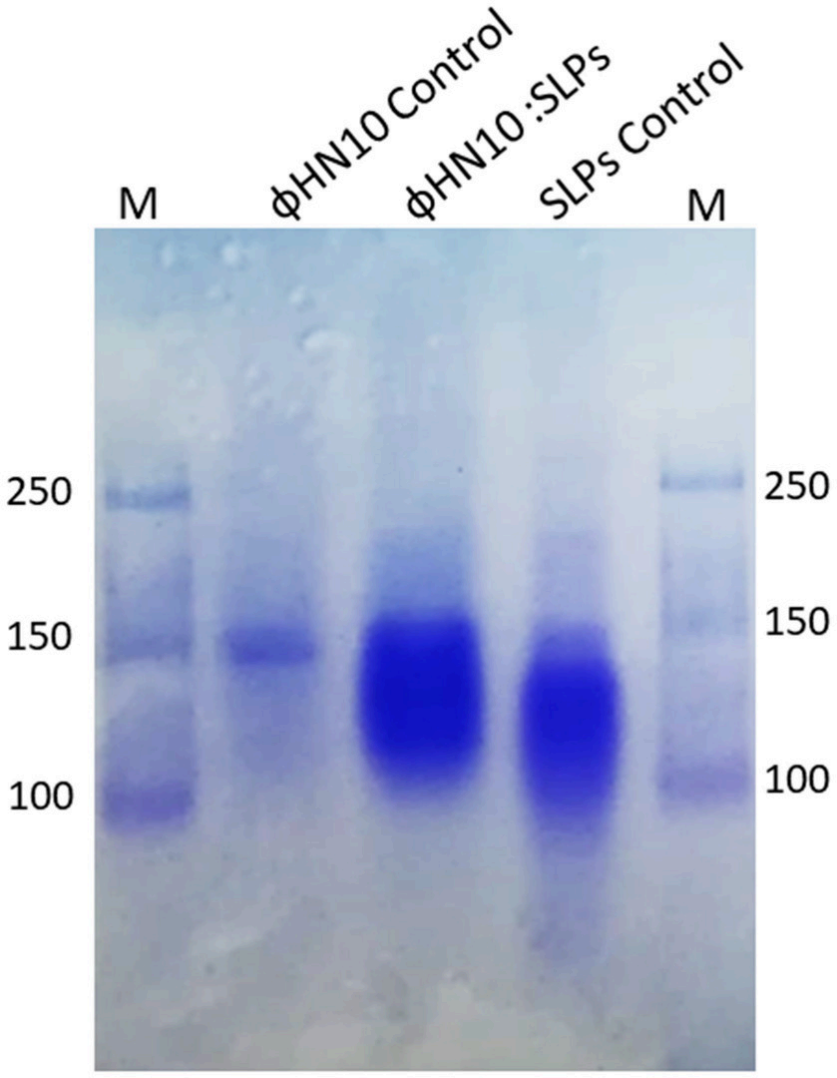
Detection of S-layer proteins (SLPs) extracted from *C. difficile* strain HN2. Native-PAGE stained with Coomassie blue; M: standard protein marker, lane labeled ΦHN10 control prepared from phage ΦHN10 alone, lane labeled ΦHN10: SLPs was SLPs after preincubation with phage for 6 h, lane labeled SLPs control was SLPs prepared from *C. difficile* strain HN2 alone.

In order to confirm the interaction between S-layer proteins and ΦHN10 phage, S-layer proteins were isolated from its susceptible host, *C. difficile* strain HN2. Analysis of the S-layer-phages interaction on the native-PAGE showed that the migration of S-layer proteins become retarded after incubating with phages when compared with the S-layer proteins alone (Figure 5). This result indicated that the S-layer protein is likely to be the molecular target on bacterial surface for phage attachment.

## Discussion

The emergence of *C. difficile* hypervirulent strains (DePestel and Aronoff, 2013) and the limiting treatment options for CDI (Venugopal and Johnson, 2012) poses an urgent threat to public health. The development of alternative anti-infection modalities has become one of the highest priorities of clinical research. Phage therapy is one of potentials treatment for CDI due to its safety and specificity toward host bacteria. However, as has been known, no strictly virulent phage that infect *C. difficile* has yet been discovered. Therefore, it is necessary to identify and characterize a range of new phages, which will provide a strong foundation of knowledge and a library of phage for further development and improvement of phages for therapeutic purposes. Here, we report the isolation and characterization of phages that target clinical strains of *C. difficile*. Although many investigators have focused on the extensive screening of *C. difficile* phages from environmental and clinical samples, there is no report on non-lysogenic phage thus far (Hargreaves and Clokie, 2014). Therefore, our work focused on inducible lysogenic *C. difficile* phages from clinical isolates. In order to reduce the massive use of inducing agents and laborious efforts from blind induction, we screened for the phage marker genes in all clinical strains in our collection using PCR. Two sets of degenerate primers targeting *C. difficile* myovirus and siphovirus *holin* genes were exploited as markers to track prophage on host genome. Of interest, prophage DNAs were detected at a very high frequency (91.8%) among clinical *C. difficile* isolates. The fact that high percentage of clinical *C. difficile* isolates harbored prophages might suggest intricate evolutionary relationship between phages with lysogenic life cycle and *C. difficile*. In fact, acquisition of many toxin genes in various toxin-producing bacteria, for example, *Staphylococcus aureus, Streptococcus pyogenes*, shiga toxin-producing *E. coli*, and *C. botulinum*, involves temperate phages (Fortier and Sekulovic, 2013).

Our results indicated that not all prophages could be induced to produce free phage particles, even though the phage specific gene was detected in their genomes. It is the evident from PCR that not only complete bacteriophage genomes but also prophage-related fragments or defective phages could be detected. Others possible explanations may be due to the lack of the optimal inducing agent and induction conditions. However, the protocol for temperate phage induction with mitomycin C is commonly used in gram-positive bacteria (Goh et al., 2005; Fortier and Moineau, 2007; Shan et al., 2012). In this study, 5 temperate phages including ΦHN10, ΦHN16-1, ΦHN16-2, ΦHN50, and ΦHR24 were successfully induced from 4 lysogenic strains with mitomycin C. The lysogenic strain HN16 harbored 2 different temperate phages, with distinguishable morphology as well as unique restriction patterns (Table 2 and Figure 2). All phages possessed a long, non-flexible, and contractile sheath tail, suggesting that they belonged to Myoviridae family of the order Caudovirales, according to ICTV classification (Mayo and Horzinek, 1998). The phages size in this study were within a size range of previously reported of *C. difficile* myovirus (Goh et al., 2005; Fortier and Moineau, 2007; Meessen-Pinard et al., 2012; Nale et al., 2012; Shan et al., 2012). Moreover, as observed by several studies of *C. difficile* phage induction, the TEM analysis also revealed the PT-LPs with T-like shape. The T-like shape resembles a contractile tail of myophage (Fortier and Moineau, 2007; Nale et al., 2012). The existence of PT-LP was also reported in other bacterial species including *Vibrio* sp., and R-type pyocin of *Pseudomonas aeruginosa* (Lee et al., 1999; Gnezda-Meijer et al., 2005). The T-like shape particle has been demonstrated to possess bactericidal activity against other bacteria of the same species and provides competitive advantage to their hosts including in *C. difficile, P. aeruginosa, Budvicia aquatica*, and *Pragia fontium* (Šmarda and Benada, 2005; Sangster et al., 2015). Since, the PT-LPs lack the full phage genomes but still have capability to kill the specific target bacteria, they could also be a promisingly safe antimicrobial agent against CDI because there will be no concern regarding horizontal gene transfer between bacteria.

Four temperate phages including ΦHN10, ΦHN16-1, ΦHN16-2, and ΦHN50 exhibited a narrow host range with the maximum number of infected host of only 6/92 isolates (6.5%) while ΦHN24 did not have any susceptible host. The narrow host range of *C. difficile* phage has also been described previously (Fortier and Moineau, 2007; Nale et al., 2012; Shan et al., 2012). Due to the lack of suitable propagating host, phages could not be replicated, posing as a major limitation for *C. difficile* phage study. There are two possible hypotheses that could explain the narrow host range of *C. difficile* phages. First, the host defense mechanisms that prevent phage infection including abortive system, restriction system, CRISPR/Cas system, and modification of phage-specific host receptor (Labrie et al., 2010; Bhaya et al., 2011). Second, the superinfection exclusion system, provided by existing prophage in the host genome, that inhibits secondary infection and infection of related phages. The narrow host range would limit its application in CDI therapy. However, four temperate phages were induced from different isolates of the ribotype 017 lysogens and this particular ribotype was sensitive to all phages as well. Moreover, the ribotype 017 is the one of the highly prevalent and virulent ribotypes worldwide beside the ribotypes 027, 078, and 014 (Cairns et al., 2012). Therefore, the identification of phage against to this ribotype will certainly impact the prevention, and the understanding of phage-host interaction of this virulent ribotype.

Stability of phages should be concerned for development of phage as therapeutic treatment. Previous studies reported that factors such as temperature and pH play important roles on the survival of phages (Pirisi, 2000; Jepson and March, 2004; Silva et al., 2014). The pH influenced attachment, infectivity, intracellular replication and multiplication of phages (Pirisi, 2000; Jepson and March, 2004). Although the optimal pH condition for long terms storage of phage is neutral pH, all of isolated phages in this study have highest infection ability in various pH values ranging from 5 to 10. From this result, efficiency of phages should not be affected by either the storage condition or the pH of human body. Temperature is also one of the important factors that destroy the infectivity of phages. The results revealed that phages show maximum stability in the temperature range of 25–37°C. Most phages, including ΦHN16-1, ΦHN16-2, and ΦHN50, are still stable up to 50°C. Similar to pH, temperature can influence the efficiency of phages and it should be chosen carefully for storage and utilization condition.

The fact that restriction endonuclease are able to cleave the genomic DNA isolated from the phage suggesting that all four temperate phages including ΦHN10, ΦHN16-1, ΦHN16-2, and ΦHN50 have dsDNA genome, which is the major genetic material type reported in the order of *Caudovirales* (tailed phage; Horgan et al., 2010; Sekulovic et al., 2014; Nale et al., 2016). The distinct restriction pattern suggest that they are all different phage particles.

Phage requires binding of specific receptor on bacterial host for initiating the infection. The phage receptor has been shown to locate at different parts of bacterial host such as the walls of both of gram-positive (Xia et al., 2011) and gram-negative (Marti et al., 2013), appendages including pili and flagella (Shin et al., 2012; Marti et al., 2013). However, the specific receptors for *C. difficile* phage have never been reported so far. Here, we focused on pili and the other cell wall appendages on the cell surface of *C. difficile* host. The mechanical shearing method was applied for preliminary identification of phage receptor on bacteria host. Typically, mechanical disruption is used for pili detachment from *E. coli* (Korhonen et al., 1980) and removing of thick cell wall of gram-positive bacteria such as *Bacillus subtilis* (Vandeventer et al., 2011). Mechanical shearing of HN2 bacterial host resulted in the decline in infectivity of ΦHN10 when compared with the normal cell host determined by spot-titer assay (Figure 3). Moreover, TEM analysis revealed that ΦHN10 attachment at mesh-like structure of bacterial cell wall, not at the pili (Figures 4C,D). Our Fourier calculation confirmed that the mesh-like structure on the cell wall surface of *C. difficile* is S-layer, containing various proteins or glycoproteins (Fagan and Fairweather, 2014). Although the results suggested that the S-layer on bacterial cell wall might be potentially a phage receptor, wall-teichoic acid (WTA) and lipoteichoic acid (LTA) could also the possible candidate receptor among the Gram-positive bacteria (Bertozzi Silva et al., 2016). Therefore, S-layer proteins were prepared and used in the phage receptor study. In this study we used the phage binding assay to investigate the cognate receptor of ΦHN10 on its host cell surface. This assay suggested that ΦHN10 can specifically bind to S-layer proteins, indicating direct interaction between the S-layer proteins to ΦHN10 phage. To our knowledge, this was the first evidence suggesting S-layer as a phage receptor of *C. difficile*. This work not only offers insights into the diversity of phages targeting *C. difficile* clinical isolates but also provide detailed information on the molecular interaction between phage and *C. difficile*.

## Author contributions

SC conceived and designed the study. WP, PO, TP, PW, and SC performed the experiments. TJ, PK, and SS helped with the experimental assays. WP, TP, and SC wrote the paper. PO and TJ edited the manuscript. SC supervised the project. All authors have read and approved the manuscript.

### Conflict of interest statement

The authors declare that the research was conducted in the absence of any commercial or financial relationships that could be construed as a potential conflict of interest.

## Supplementary material

Supplementary Figure 1 Prophage carriage *C. difficile* detection. Gel electrophoresis revealed representative PCR products of *holin* gene in *C. difficile* isolates. **(A,B)** shown *holin* gene of myovirus and siphovirus, respectively.
