# Supplementary figures and images for: Characterization of Bacteriophages Infecting Clinical Isolates of Clostridium difficile
Source: Front Microbiol. 2018 Jul 31;9:1701. doi: 10.3389/fmicb.2018.01701 (PMC6079236; doi:10.3389/fmicb.2018.01701)

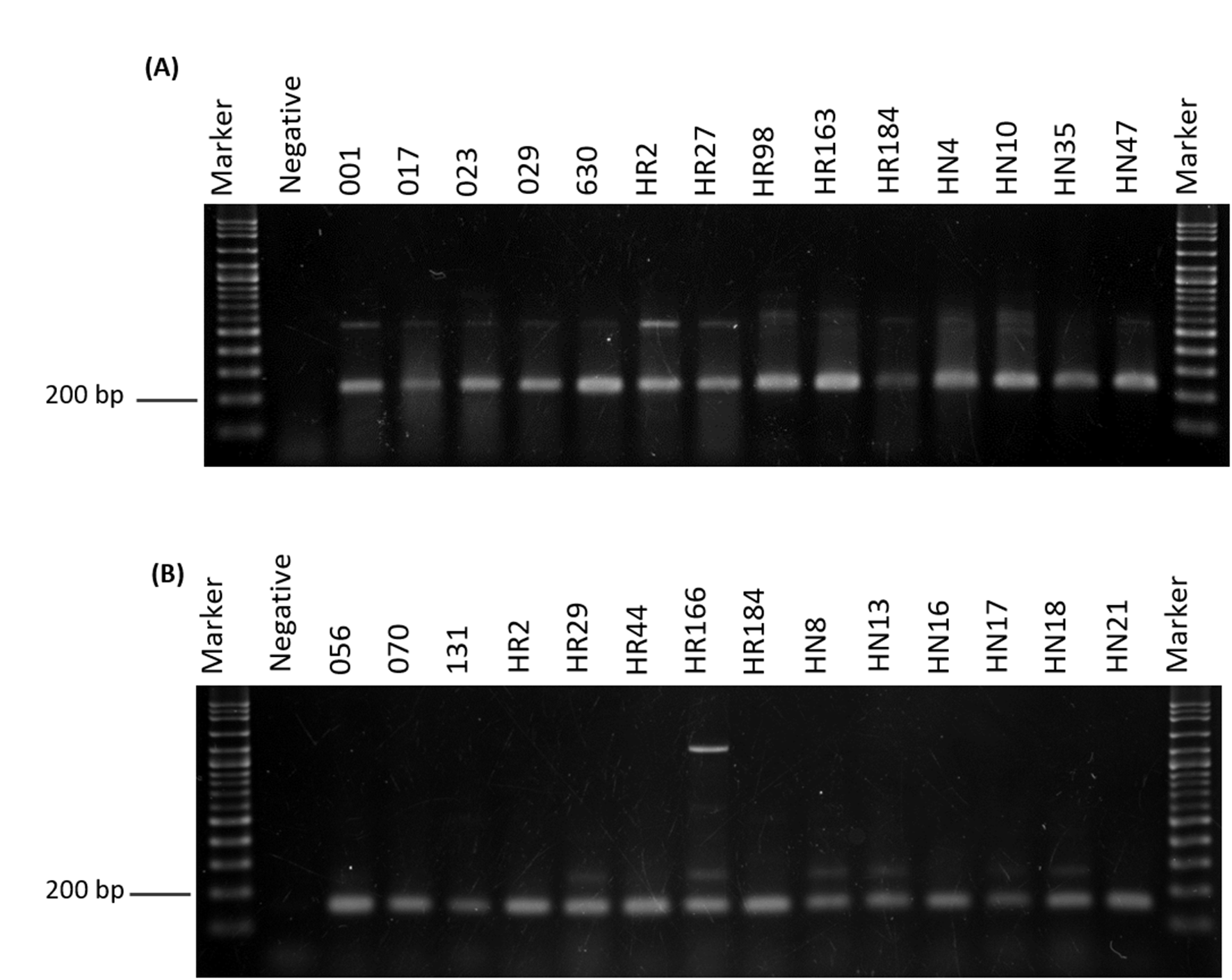

Supplement: Supplementary Figure 1 — Prophage carriage C. difficile detection. Gel electrophoresis revealed representative PCR products of holin gene in C. difficile isolates. (A,B) shown holin gene of myovirus and siphovirus, respectively. [file Image_1.TIF]
